# Entropy Information of Cardiorespiratory Dynamics in Neonates during Sleep

**DOI:** 10.3390/e19050225

**Published:** 2017-05-15

**Authors:** Maristella Lucchini, Nicolò Pini, William P. Fifer, Nina Burtchen, Maria G. Signorini

**Affiliations:** 1 Department of Psychiatry, Columbia University College of Physicians & Surgeons, New York, NY 10032, USA; 2 Dipartimento di Elettronica, Informazione e Bioingegneria, Politecnico di Milano, 20133 Milano, Italy; 3 Department of Psychosomatic Medicine and Psychotherapy, University of Freiburg, 79106 Freiburg, Germany

**Keywords:** newborn, sleep state, autonomic nervous system, heart rate variability, transfer entropy

## Abstract

Sleep is a central activity in human adults and characterizes most of the newborn infant life. During sleep, autonomic control acts to modulate heart rate variability (HRV) and respiration. Mechanisms underlying cardiorespiratory interactions in different sleep states have been studied but are not yet fully understood. Signal processing approaches have focused on cardiorespiratory analysis to elucidate this co-regulation. This manuscript proposes to analyze heart rate (HR), respiratory variability and their interrelationship in newborn infants to characterize cardiorespiratory interactions in different sleep states (active vs. quiet). We are searching for indices that could detect regulation alteration or malfunction, potentially leading to infant distress. We have analyzed inter-beat (RR) interval series and respiration in a population of 151 newborns, and followed up with 33 at 1 month of age. RR interval series were obtained by recognizing peaks of the QRS complex in the electrocardiogram (ECG), corresponding to the ventricles depolarization. Univariate time domain, frequency domain and entropy measures were applied. In addition, Transfer Entropy was considered as a bivariate approach able to quantify the bidirectional information flow from one signal (respiration) to another (RR series). Results confirm the validity of the proposed approach. Overall, HRV is higher in active sleep, while high frequency (HF) power characterizes more quiet sleep. Entropy analysis provides higher indices for SampEn and Quadratic Sample entropy (QSE) in quiet sleep. Transfer Entropy values were higher in quiet sleep and point to a major influence of respiration on the RR series. At 1 month of age, time domain parameters show an increase in HR and a decrease in variability. No entropy differences were found across ages. The parameters employed in this study help to quantify the potential for infants to adapt their cardiorespiratory responses as they mature. Thus, they could be useful as early markers of risk for infant cardiorespiratory vulnerabilities.

## 1. Introduction

Sleep in humans is characterized by the activation of numerous cortical, subcortical and medullar neural circuits, which require complex co-ordination during sleep, regulated by hormonal changes, cardiovascular challenges, circadian variations and other factors [1]. The autonomic nervous system (ANS) is a central actor in the regulation of sleep physiology, modulating cardiovascular functions during the onset of sleep and transitions to different sleep states.

Heart rate variability (HRV) is a non-invasive and reliable measure of autonomic function, and is ideally suited for probing changes in cardiovascular autonomic control. A vast literature demonstrates the utility of HRV measurement with several different analytic approaches across many populations [2]. These approaches are particularly useful in the assessment of newborn infants who are very immature and for whom standard protocols designed for adults requiring cooperation are inappropriate. The application of a wide range of different approaches, such as linear and non-linear methods during different stages of sleep, has allowed for the direct quantification as well as a better understanding of the physiological autonomic changes that are sleep state-dependent [3].

In newborns previous studies based on traditional time and frequency domain analysis of HRV have reported that HR and overall HRV were lowest in quiet sleep. The highest values were found in the waking state [4]. This has been ascribed to more variable respiratory patterns and sympathetic predominance in the waking/active state [5].

Entropy measures, such as Approximate or Sample Entropy, for assessing regularity/complexity in a time series have emerged as powerful tools for HRV analysis, since they provide entropy estimation indices without making any assumptions about the underlying structure of the system [6,7]. Interestingly, a decrease in the Sample Entropy value was reported following a positional change from supine to standing in human adults. This result was associated with a reduction of vagal influence on the heart paired with sympathetic activation [8,9]. Thus, almost unexpectedly, a more complex dynamic of the human sympathovagal balance can emerge in a resting or relatively inactive state. This relationship between entropy values and the sympathovagal balance is also supported by animal studies [10,11].

Similar findings have shown that non Rapid Eye Movement (NREM) sleep, referred to as quiet sleep in infants, is characterized by an increase in Sample Entropy. Results were less definitive in Rapid Eye Movement (REM)/active sleep [12–14].

Furthermore, the cardiovascular and respiratory systems are governed by complex mutual interactions that may require alternate analytic techniques. Such approaches have ranged from cross-spectral analysis to non-linear methods, e.g., mutual information [15–17], considering linear and nonlinear relations between HR and respiration signal.

Nonetheless, a limitation of all the aforementioned techniques is that they do not measure directional relationships, and thus, the features they provide can only partially reveal the underlying interacting mechanisms responsible for the changes in complexity, especially when knowledge of the underlying physiology is limited.

Transfer Entropy (TE) was developed to precisely address this issue. Its focus is on tracking the information flow between two systems. Specifically, TE can enhance the quantification of the directional coupling between respiration and HR in order to incorporate both sympathetic and parasympathetic regulatory influences [18]. The main advantages of this method are that it captures both linear and nonlinear contributions to information flow, and it can differentiate the directionality of transfer.

This manuscript will present analyses of data collected on a population of 151 newborn infants. A subset (*n* = 33) was also followed up at 1 month of age. The study was designed to first apply traditional methods of signal processing to HR and respiration signals. The application of non-linear techniques was then employed to provide an additional layer of analysis to uncover more complex cardiorespiratory interactions. The aim was to combine information coming from different domains to better understand the physiology underlying cardiorespiratory regulation during sleep and to show how novel nonlinear methods can complement traditional approaches.

Furthermore, an enhanced understanding of the cardiorespiratory regulatory mechanisms during sleep in infants might provide us with better tools to assess risk for abnormal development of autonomic dysfunction and cardiorespiratory dysregulation.

## 2. Materials and Methods

### 2.1. Patients and Data Collection

The analysis presented in this paper is from data acquired on 151 newborns (gestational age at birth 38–40 weeks). Out of these selected infants, 33 came back for a 1 month follow-up.

None of the infants enrolled had been admitted to the Neonatal Intensive Care Unit nor had any major illness, congenital abnormalities or known genetic disorders. Mothers were at least 18 years of age and displayed no evidence of major illness or psychiatric disorders during the pregnancy.

ECG and respiratory activity were recorded non-invasively at a sampling rate of 500 Hz and 200 Hz, respectively, by means of three leads on the chest in standard positions (RA, RL, LL, DATAQ Instruments) and by a respiratory inductance belt around the infant abdomen (Ambulatory Monitoring Inc., Ardsley, NY, USA).

During the acquisition infants were sleeping supine and sleep states were classified into active sleep (AS) and quiet sleep (QS) by an expert clinician [19].

Segments of 300 consecutive beats in the same sleep state were identified and then analyzed: the total number of segments was 525 (304 AS, 221 QS) for newborns and 247 (108 AS, 139 QS) for 1-month-old infants. The average length of the 300 beats segments was 148.30 ± 14.01 s for newborns in AS, 151.63 ± 12.29 s for newborns in QS, 124.02 ± 8.55 s for 1-month-old infants in AS and 129.87 ± 10.04 s 1-month-old infants in QS.

The length of the segments was chosen based on previous studies, which showed that 300 beats was an appropriate number for Transfer Entropy estimation [20]. Moreover, for time domain and frequency domain measures in adult subjects, the suggested approach is to employ segments of 5 min [2]. Given that the average HR of infants is double that of adults, analyses were computed using 300 infant beats.

Study protocols were performed at Columbia University Medical Center, upon mothers’ consent and approval of the Institutional Review Board of the New York State Psychiatric Institute and of the Columbia University Medical Center.

### 2.2. Signal Processing

R wave peaks were detected on the ECG with the Pan-Tompkins algorithm [21]. An adaptive filter was then applied to remove ectopic beats or artifacts, preserving the beat-to beat sequence.

Respiratory signals were band-pass filtered from 0.05 to 3.5 Hz. Peaks of inspiration/expiration were identified in order to create the Inter Breath Interval series (IBI). Afterwards, the respiratory signal was sampled at times synchronous with the R peaks previously identified, in order to define two simultaneous events.

Time domain parameters proposed in the Task Force of 1995 for RR series analysis were estimated [2], including mean RR, Standard Deviation of Normal to Normal RR intervals (SDNN) and Root Mean Square of the Successive Differences (RMSSD).

For the respiratory signal, mean IBI and IBI inter quartile range (IQR) were calculated.

RR series and respiration were both resampled at 5 Hz to assure that the two signals were synchronous in time. Power Spectral Density (PSD) of RR series was calculated with a parametric approach based on Auto Regressive (AR) modelling. The AR model order was fixed at 10. Automatic decomposition of signal power (variance) provided the automatic quantification of different frequency components, each one identified by the central frequency and amount of power associated to that specific component.

In order to partition the total variance into frequency components, Very Low Frequency (VLF), 0.01–0.04 Hz, Low Frequency (LF), 0.04–0.2 Hz, and High Frequency (HF), 0.35–1.5 Hz band values were specifically chosen for this population of infants.

Areas under the curve for HF and LF frequency components were calculated and presented as percentages of the total power, excluding VLF. As the literature confirms, LF and HF reflect both to sympathetic and parasympathetic influences on ANS control, and their balance is fundamental for understanding cardiorespiratory regulation processes [22].

A different approach, evaluating the rate of regularity/irregularity of HR, was employed using estimates of the Sample Entropy (SampEn) index. SampEn, as proposed by Richman and Moorman [6], was adopted to obtain a measure of RR series complexity. SampEn computation used parameters values *m* = 1, 2, 3 and *r* = 0.2 × std (of the considered signal). Another estimator of signal entropy information was calculated: the Quadratic Sample entropy (QSE) index has been presented by Lake as an improvement of SampEn. [23,24].

The choice of *m* was identical to SampEn estimation, while the *r* parameter changed based on the minimum numerator count method, which increments *r* in order to obtain a minimum number of matches *N* to increase the reliability of the estimate. Our calculation considered *N* = *n*^2^/5, with *n* equal to the length of the signal (300 samples).

#### Transfer Entropy

The Transfer Entropy index evaluates the information flow from a source system *X* to a destination system *Y*, considered as two interacting dynamical subsystems.

*X_n_*, *Y_n_* are the stochastic variables obtained by sampling the stochastic processes, describing the state visited by the systems *X* and *Y* over time.

Furthermore, $Y_{n}^{-},\;X_{n}^{-}$ are the vector variables representing the entire history of the processes.

The Transfer Entropy from *X* to *Y* is defined as:

(1) $${\text{TE}}_{X\to Y}=\sum p(Y_{n},Y_{n}^{-},X_{n}^{-})\log\frac{p(Y_{n}|Y_{n}^{-},X_{n}^{-})}{p(Y_{n}|Y_{n}^{-})}$$

From this definition, it emerges that TE can be also expressed as a difference of two conditional entropies (CE):

(2) $${\text{TE}}_{X\to Y}=H(Y_{n}|Y_{n}^{-})-H(Y_{n}|Y_{n}^{-},X_{n}^{-})$$

In other words, TE quantifies the information provided by the past of the process *X* about the present of the process *Y*, that is not already provided by the past of *Y*.

TE parameter is a powerful tool to detect information transfer given that it does not require any particular model assumption describing the interactions regulating the system dynamics. Moreover, it is able to uncover purely non-linear interactions and to deal with a range of interaction delays.

Nevertheless, the TE method requires the approximation of the infinite-dimension variables representing the past of the processes. In the following, we will briefly clarify this issue. More specifically, a reconstruction of the past of the system dynamics is represented by the processes *X* and *Y* with reference to the present state of the destination process *Y*. This allows for the acquirement of a vector $V=[V_{n}^{Y},\;V_{n}^{X}]$ containing the most significant past variables to explain the present of the destination system.

Two approaches can be applied: uniform and non-uniform embedding schemes.

In the first case (uniform), components to be included in the embedding vectors are selected a priori and separately for each time series. For example, the vector $Y_{n}^{-}$ is approximated using the embedding vector $V_{n}^{Y}=[Y_{n-m},\;Y_{n-2m}\ldots Y_{n-dm}]$, where *d* and *m* are the embedding dimension and embedding delay, respectively.

Following this approach, TE estimation consists of two steps: collection of past states of the process and estimation of entropy, with a chosen estimator. The main limitation in this case stems from the arbitrariness and potential redundancy of the estimate, which may cause problems such as overfitting and the detection of false influences. For tests of significance employed with this approach, please refer to Reference [20].

The alternative strategy is to apply non-uniform embedding. This technique consists of a progressive selection among the available variables describing the past of the observed processes *X*, *Y*, considered up to a maximum lag, and to identify the most informative variable for the destination variable *Y_n_*.

Thus, a criterion for maximum relevance and minimum redundancy is applied for candidate selection, and the resulting embedding vector ***V*** includes only the components of $\;X_{n}^{-}$ and $Y_{n}^{-}$, which contribute most to the description of *Y_n_*. In this way, no a priori choice of embedding dimension *d* is needed. In contrast to the classical embedding dimension estimation [25], in this application *d* dimension can range from 1 up to 10 and it is optimized for each estimation.

Moreover, the variables included into the embedding vector are associated by definition with a statistically significant contribution to the description of *Y*. Thus, the statistical significance of the TE estimated with non-uniform embedding emerges from the selection of at least one past component of the source process. Otherwise, the estimated TE will be zero and nonsignificant.

Another crucial aspect of the TE method is the choice of the appropriate method to estimate the joint probability distribution capable of fully describing the interrelationship between *X* and *Y*, in order to estimate the two conditional entropies needed to obtain the TE value.

The first approach adopts the linear estimator (LIN), assuming that the overall process has a joint Gaussian distribution. Under this assumption, the two CE terms defining the TE can be quantified by means of linear regressions involving variables taken from the embedding vector, depending on which embedding method is used (all variables or the relevant ones only).

The second estimator is based on a fixed state space partitioning (BIN), which consists of a uniform quantization of the time series. Then, entropies are computed by approximating probability distributions with the frequencies of occurrence of the quantized states.

The third estimator is based on the K-Nearest Neighbor technique (NN), a powerful non-parametric technique for classification, density and regression estimation. It estimates entropy terms through a neighbor search in the space defined by all the variables.

A potential problem with uniform and non-uniform embedding procedures relates to the issue of dimensionality. Adopting the non-uniform embedding could overcome this potential. As a matter of fact, this method reduces the candidates of significant past states, preventing the risk of probability density function to assume a constant value.

This non-uniform embedding method was chosen in combination with the nearest neighbor entropy estimator, since this arrangement was suggested to have high sensitivity and specificity both for linear and non-linear systems [20].

Previous publications have tested the TE method on simulated data, such as spatiotemporal systems (tent map or Ulam map) [18], verifying that the method successfully quantified information flow, with a range of estimation techniques, embedding approaches and segment lengths [20].

The MuTE toolbox was employed to estimate Transfer Entropy values. A detailed description of the methods can be found in Montalto et al. [20].

**Figure 1:**
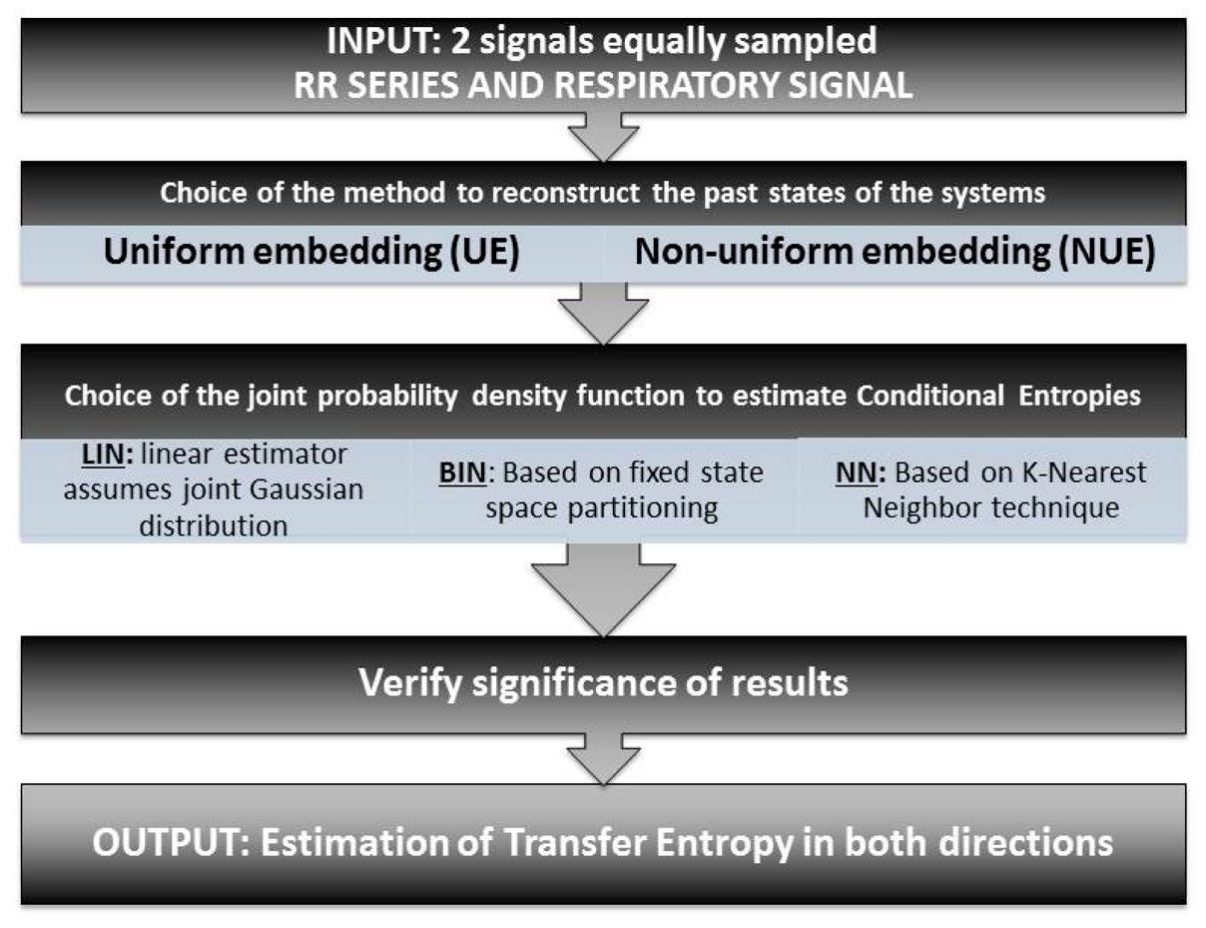
Scheme of the main steps involved in TE calculation: (1) Selection of the two signals of interest equally spaced. TE will be calculated evaluating the directionality of signal 1 → signal 2 and vice versa. (2) Choice of the method to approximate the infinite-dimension past states of the systems (UE vs. NUE). (3) Choice of Conditional Entropy estimator (LIN vs. BIN vs. NN) and TE estimation. (4) Verification of TE results significance.

The main steps for TE estimation procedure are summarized in Figure 1.

### 2.3. Statistical Analysis

After computation of HR and coupling parameters, outlier rejection of parameters was performed utilizing the Inter-Quantile Range method. TE differences between quiet and active sleep were tested, separately for each directionality. Normally distributed variables were tested with an unpaired *T*-test (Lilliefors normality test), while non-normal distributions were tested with a Wilcoxon signed-rank test.

## 3. Results

Time domain parameters confirmed previous findings, showing significantly higher overall variability (SDNN) in infants during AS, at both time points. Short term variability (RMSSD) did not differ by state at the newborn stage nor at the 1-month follow-up. Respiration was slower and less variable in QS, as expected.

Regarding change in time domain parameters with age, an increase in HR with a corresponding decrease in variability was observed in both states. Respiration rate, but not variability, changed with age, as mean IBI in QS increased with age. Time domain parameters are presented in Table 1.

Power spectra analyses showed that variance is distributed differently across bands depending on sleep state, as presented in Table 1. In QS, the percentage of power in the HF band is significantly higher than in AS, and the percentage of power in the LF band during QS is lower than during AS in the newborn population.

Regarding the RR series entropy values, both SampEn and QSE shown in Table 1 confirmed previous findings by our group, with lower values of entropy in QS for all *m* and at both ages. None of these values change significantly with age.

**Figure 2:**
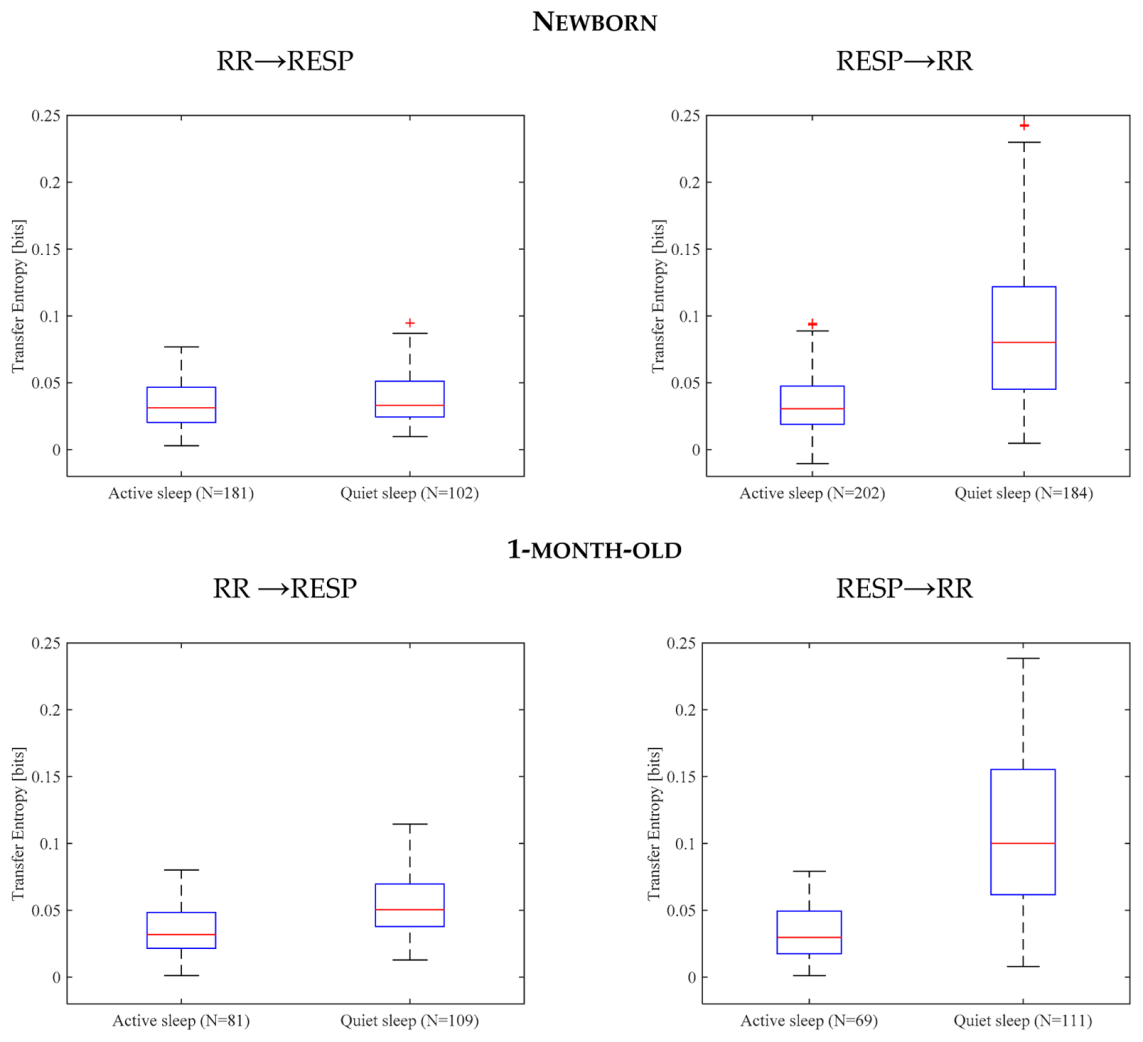
Boxplot of TE values. In the first row are shown values for newborn infants in active and quiet sleep with directionality RR → RESP on the left and RESP → RR on the right. In the second row, the same information is presented for 1-month-old infants.

At both ages, TE in QS was significantly higher than in AS, both with respect to RESP → RR and RR →RESP, as shown in Figure 2.

Comparing the two directionalities, there was no clear difference in AS, while in QS RESP → RR directionality was clearly dominant over RR → RESP, both for newborn and 1-month-old infants (Table 1).

With respect to the development of TE in the first month of life, as seen in Table 1, the major differences occur in QS, with an increase in information flow in both directions with age, while in AS values remain unchanged.

## 4. Discussion

For healthy full-term newborns, sleep constitutes the predominant state, with active sleep occurring the most frequently. The autonomic nervous system is modulated by sleep state dynamics affecting cardiorespiratory patterns. Investigation of the functional organization of these neurophysiological systems is extremely challenging, due to their intrinsic complexity and the necessity to employ noninvasive observation only. Fortunately, analysis of HRV, breathing and their coupling provide an optimal set of noninvasive functional probes of the behavior of cardiorespiratory systems.

This paper provides a comparison of traditional and novel techniques to characterize cardiorespiratory behavior during sleep in young infants at birth and at 1 month of age. The major innovation consists of the application of a parameter called Transfer Entropy to quantify the information flow between RR series and respiration and vice versa.

Only time domain parameters from this data analysis reveal a change over time, with an increase of mean HR and a decrease of HRV, while frequency domain and entropy comparisons were not significant. This could be related to the fact that 1 month of age is a transitional phase for autonomic balance going from early life to later infancy and childhood. A temporary peak in HR is often observed, while after the first month HR will gradually decrease and HRV will increase as vagal regulation matures under normal conditions [14,26]. Thus, this extremely variable sympathetic-parasympathetic equilibrium at 1 month of age could explain why measures in the frequency domain and entropies did not detect a significant change in the first month of life.

In regard to the comparison between sleep states, time domain parameters indicate an increase in overall variability in AS (SDNN), while no difference was found in beat-to-beat variability (RMSSD) both at birth and at 1 month of age. To better identify the sources responsible for these changes in variability, power spectral estimation showed that RR variance is distributed differently in HF and LF frequency bands depending on sleep states. HF has the major contribution in QS. These results combined with time domain parameters suggest increased parasympathetic activation in QS [2].

SampEn and QSE indices presented higher values in QS, and this further indicates the predominance of parasympathetic control in QS. This is convergent with other studies which proposed that a simplification of HR dynamics, and thus a lowering in entropy values, might follow parasympathetic withdrawal and sympathetic activation [8,9].

Our results also converge with previous results by Pincus et al., who found higher values of approximated entropy (ApEn) in QS (less regular) with respect to AS [27], and with another measure of complexity based on Mutual Information, AIF [15]. Additionally, TE results illustrate that QS is a state in which information flow between HR and respiration is higher than in AS. Given that SampEn and QSE should provide a measure of complexity of a signal, higher entropy values in QS could be associated with an increased interaction with other physiological systems. These findings suggest that cardiorespiratory interactions in QS transfer more information than in AS in healthy infants, as confirmed by Frasch et al. [15].

In QS, coupling is stronger for both information flow directions. Moreover, the main direction of the information flow is RESP → RR in QS, while in AS this not evident in both newborn and 1-month-old infants.

As highlighted in previous work [28], for infants the directionality of cardiorespiratory reciprocal interaction is not the same as in adults, with phenomena like respiratory sinus arrhythmia. Nonetheless, our results seem to indicate a difference in directionality balance based on sleep state. This could be driven by differences in the average breathing frequency. i.e., when breathing frequency is higher there is less opportunity for the respiration to dynamically modulate HR. Higher breathing frequency occurs more often in AS, and this could account for an absence of a dominant directionality.

An age-dependent change in information flow happens only in QS. AS is per se a state of lower coupling between HR and respiration and this does not dramatically change with age. Further investigation in older infants is warranted.

From a methodological point of view, entropy measures tend to be influenced by the choice of the values for parameters *r*, *m*, *N*, as previously reported [29]. In our study, the consistency of entropy measures can be observed independent of the parameter choice. In fact, our results were not affected by parameter *m*. In preliminary studies, we also tested the same measures on segments with *N* equal to 100 and 200 beats and used 3 min epochs, and obtained comparable results. This strongly suggests that the difference between groups remains stable.

One limitation of this study is that the HR and respiration interactions can operate across multiple time lags. Thus, rates of information production and exchange can vary with the temporal relationships between signals. This issue can be more fully addressed with systematic analyses, incorporating TE estimation of interactions over multiple time scales.

In sum, we have provided a quantification of sleep state differences in cardiorespiratory regulation combining both linear and complexity parameters. In QS, the nervous system seems to adopt a more complex organization, which might extend to a wide range of behavioral states in which HR and respiration interact in more complex ways. This would construct a highly adaptable and flexible system for unpredictable and ever-changing environments. Information flow between HR and respiration is increased in QS with respect to AS, indicating that more information is exchanged between the cardiac and the respiratory systems.

This approach to the quantification of cardiorespiratory interactions affords the potential for early assessment of infant development of bidirectional control between physiological systems. Finally, the proposed approach could facilitate early risk assessment for neurophysiological disorders such as sudden infant death syndrome (SIDS) [30].

## Figures and Tables

**Table 1:** Parameter values (mean ± SD) for 300-beat segments, for newborn and 1-month-old infants in active and quiet sleep. *p*-values indicate statistical comparison between active and quiet sleep for newborn and 1-month-old infants, and between newborn and 1-month-old infants in active and quiet sleep.

Parameter	Parameter Values				*p*-Values			

	NB		OM		AS vs. QS		NB vs. OM	

	AS	QS	AS	QS	NB	OM	AS	QS
**Time domain**								
RR mean [s]	0.496 *±* 0.047	0.509 *±* 0.039	0.415 *±* 0.029	0.425 *±* 0.022	n.s.	n.s.	<0.01	<0.01
SDNN [ms]	34.028 *±* 13.940	25.982 *±* 10.140	23.168 *±* 6.567	15.334 *±* 6.008	<0.01	<0.01	<0.01	<0.01
RMSSD [ms]	17.382 *±* 8.869	18.165 *±* 7.815	11.730 *±* 3.896	10.050 *±* 4.924	n.s.	n.s.	<0.01	<0.01
IBI mean [s]	1.240 *±* 0.260	1.483 *±* 0.264	1.315 *±* 0.240	1.630 *±* 0.340	<0.01	<0.01	n.s.	<0.05
IBI IQR [s]	0.440 *±* 0.146	0.258 *±* 0.088	0.404 *±* 0.144	0.278 *±* 0.102	<0.01	<0.01	n.s.	n.s.

**Frequency domain**								
LF/(LF + HF) [%]	0.891 *±* 0.065	0.770 *±* 0.125	0.873 *±* 0.102	0.7745 *±* 0.177	<0.01	<0.05	n.s	n.s.
HF/(LF + HF) [%]	0.101 *±* 0.056	0.230 *±* 0.125	0.116 *±* 0.091	0.195 *±* 0.143	<0.01	n.s.	n.s	n.s.

**Conventional entropies**								
SampEn1 [bits]	1.76 *±* 0.26	1.98 *±* 0.14	1.69 *±* 0.28	1.94 *±* 0.15	<0.01	<0.01	n.s.	n.s.
SampEn2 [bits]	1.63 *±* 0.31	1.84 *±* 0.19	1.58 *±* 0.30	1.86 *±* 0.16	<0.01	<0.01	n.s.	n.s.
SampEn3 [bits]	1.52 *±* 0.37	1.69 *±* 0.25	1.49 *±* 0.35	1.74 *±* 0.24	<0.01	<0.01	n.s.	n.s.
QSE1 [bits]	7.89 *±* 0.21	8.05 *±* 0.16	7.82 *±* 0.25	8.04 *±* 0.18	<0.01	<0.05	n.s.	n.s.
QSE2 [bits]	7.92 *±* 0.21	8.07 *±* 0.15	7.87 *±* 0.25	8.09 *±* 0.14	<0.01	<0.01	n.s.	n.s.
QSE3 [bits]	7.97 *±* 0.21	8.11 *±* 0.14	7.91 *±* 0.26	8.12 *±* 0.15	<0.01	<0.01	n.s.	n.s.

**Transfer entropy**								
RR → RESP [bits]	0.03 *±* 0.02	0.04 *±* 0.02	0.04 *±* 0.02	0.06 *±* 0.02	< 0.05	< 0.01	n.s.	< 0.01
RESP → RR [bits]	0.04 *±* 0.02	0.09 *±* 0.06	0.03 *±* 0.02	0.10 *±* 0.06	< 0.01	< 0.01	n.s.	< 0.05

NB: newborns, OM: 1-month-old infants, AS: active sleep, QS: quiet sleep. SDNN: standard deviation of normal to normal RR intervals, RMSSD: root mean square of the successive differences, IQR: inter-quantile range, IBI: inter-breath interval, LF: low frequency, HF: high frequency, SampEn: sample entropy, QSE: quadratic sample entropy.
